# Malignant gastrointestinal neuroectodermal tumor presenting with small intestinal obstruction: A case report

**DOI:** 10.1002/deo2.119

**Published:** 2022-04-10

**Authors:** Makiko Sasaki, Mamoru Tanaka, Koki Asukai, Hiroki Koguchi, Yusuke Inoue, Mizuki Moriyama, Tetsuo Tsukahara, Takeo Kawahara, Eiji Hayashi, Yukinori Hattori, Izumi Hasegawa, Hiromi Kataoka

**Affiliations:** ^1^ Department of Gastroenterology Japan Community Health Care Organization Chukyo Hospital Aichi Japan; ^2^ Department of Gastroenterology and Metabolism Nagoya City University Graduate School of Medical Science Aichi Japan; ^3^ Department of Surgery Japan Community Health Care Organization Chukyo Hospital Aichi Japan; ^4^ Department of Surgery Kasugai Municipal Hospital Aichi Japan; ^5^ Department of Surgery Anjo Kosei Hospital Aichi Japan; ^6^ Department of Pathology Japan Community Health Care Organization Chukyo Hospital Aichi Japan

**Keywords:** balloon enteroscopy, clear cell sarcoma, intestinal obstruction, neuroectodermal tumor, small intestine

## Abstract

Malignant gastrointestinal neuroectodermal tumors (GNETs) are rare malignant mesenchymal neoplasms. To our knowledge, only 99 cases have been reported worldwide. The tumor has an aggressive malignancy, with a rapid progression. The histological features of GNET overlap with those of clear cell sarcoma, which contain Ewing sarcoma breakpoint region 1 mutation. GNETs lack melanocyte‐specific markers, while clear cell sarcoma exhibits melanocytic differentiation. Various symptoms have been reported previously, and the most reported lesion is in the small bowel. The patient was a 69‐year‐old man who presented with abdominal pain and vomiting. Computed tomography revealed a nodule in the small bowel, which induced small intestinal obstruction. Enteroscopic images revealed a submucosal tumor. Surgery was performed, and the patient was diagnosed with GNET. Only two patients whose primary lesions were in the small intestine, including the patient in this report, have undergone enteroscopy before surgery. This is a rare case of GNET in which a patient underwent enteroscopy before surgical treatment.

## INTRODUCTION

Malignant gastrointestinal neuroectodermal tumor (GNET) was previously called clear cell sarcoma‐like gastrointestinal tumor (CCSLGT) because it resembles clear cell sarcoma (CCS) on microscopy. After the accumulation of similar cases and investigations, tumors with these characteristics were designated as GNETs.^1^ GNETs are rare tumors that are occasionally difficult to diagnose. GNETs should be diagnosed correctly because of their aggressive malignancy and rapid progression, but the rarity of this disease makes it difficult to be stated as a differential diagnosis case. Herein, we describe a rare case of GNET in which the patient underwent enteroscopy before surgery and discuss the advantages of endoscopy.

## CASE REPORT

**FIGURE 1:**
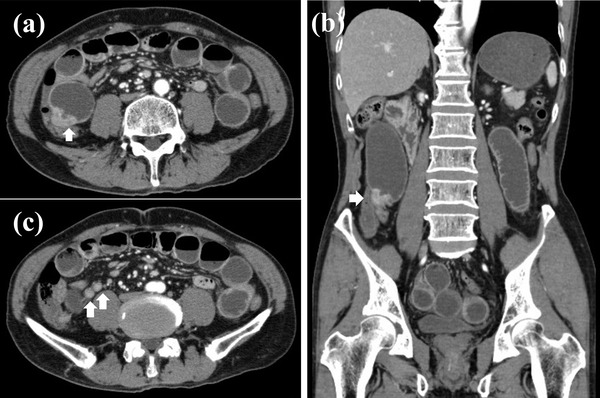
Abdominal computed tomography findings. (a,b) The tumor caused intestinal obstruction (c) accompanied by enlarged lymph nodes. White arrows in (a) and (b) indicate the main lesion, and those in (c) indicate the swollen lymph nodes

**FIGURE 2:**
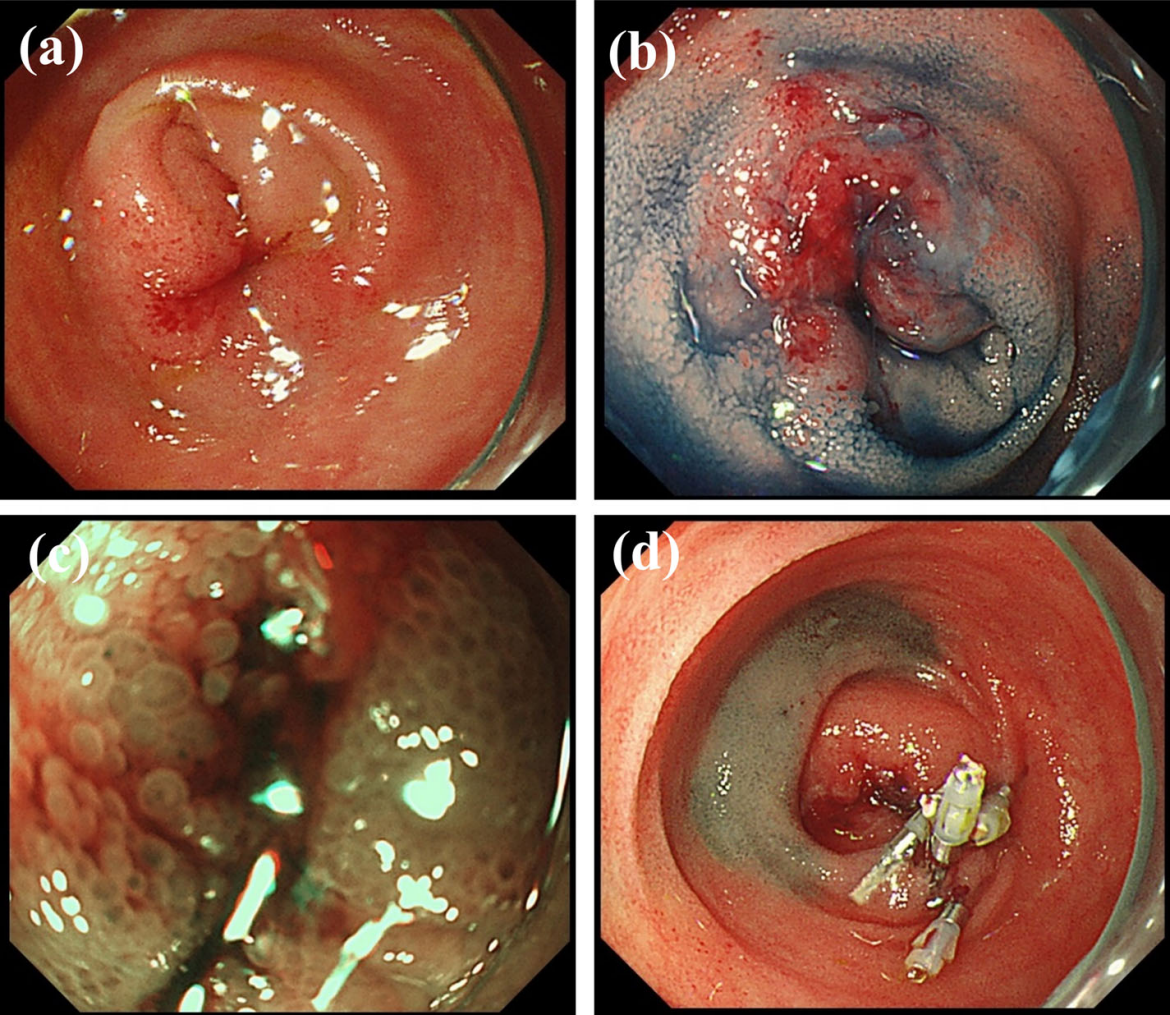
Findings of single‐balloon enteroscopy. (a–c) The primary circumferential tumor covered with non‐neoplastic epithelium. (d) The occlusion site was marked by the clip‐marking and Indian ink‐injection methods

**FIGURE 3:**
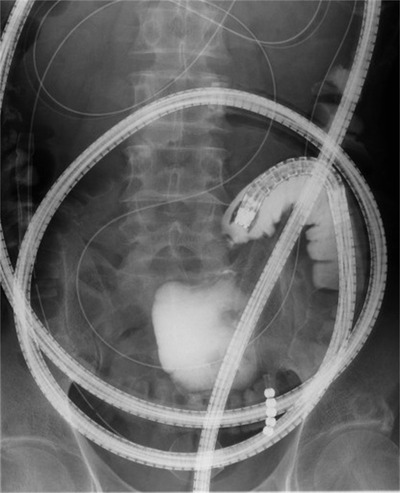
Roentgenography showing obstruction by a tumor. The process was performed using enteroscopy under fluoroscopic guidance

**FIGURE 4:**
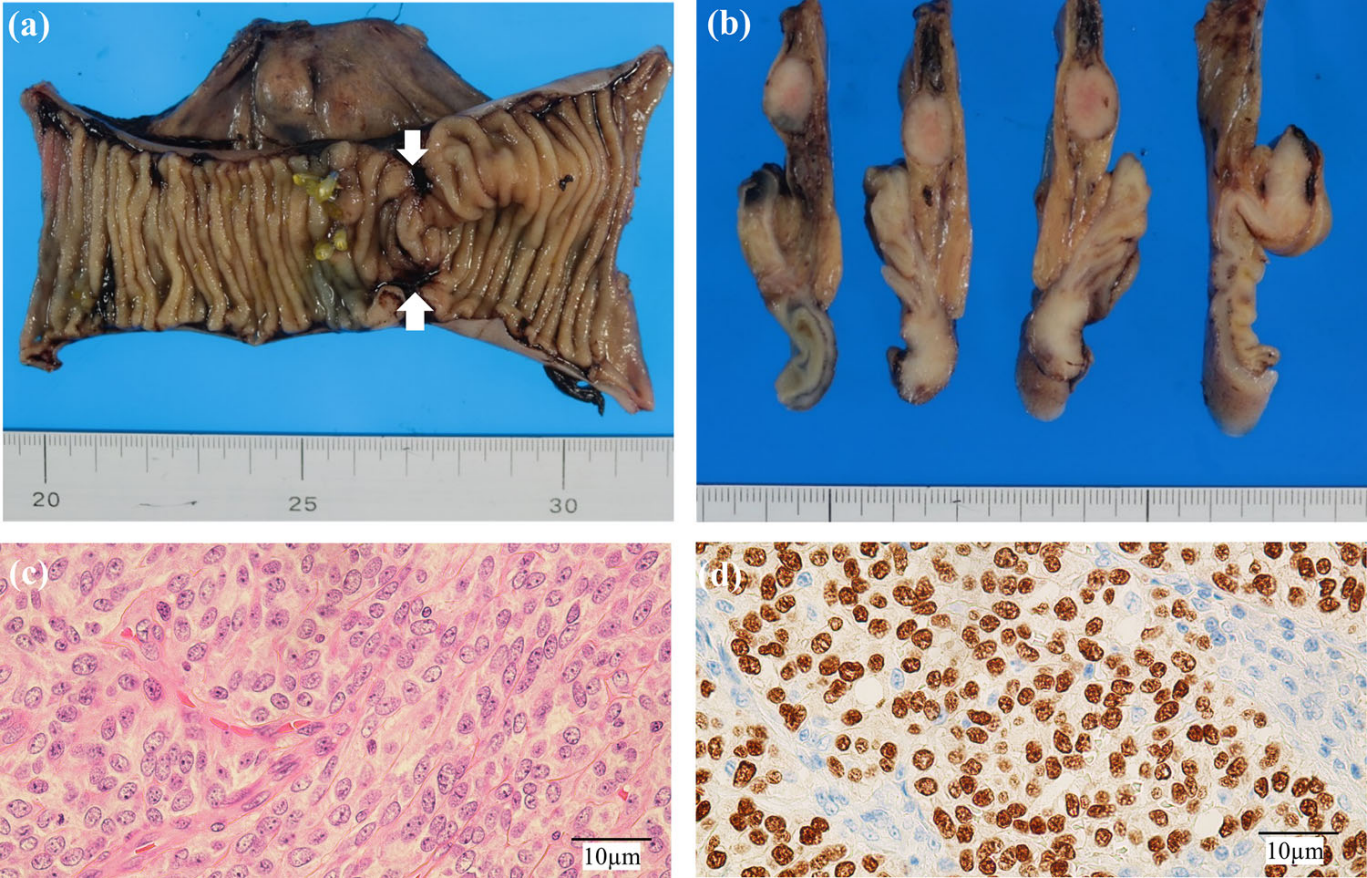
Surgically resected specimen (jejunum). (a) Macroscopic view of the whole specimen. (b) Sectioned specimen. (c) Hematoxylin and eosin staining (x400). (d) SOX‐10 staining (x400)

A 69‐years‐old man was referred by a family physician to the emergency department for the management of abdominal pain with vomiting. The patient had no significant family history, surgical history, or allergies. There was neither a history of smoking nor other habits of medical importance. On physical examination, the patient's temperature was 36.8°C, heart rate was 70 beats per minute, respiratory rate was 15 breaths per minute, and blood pressure was 135/72 mmHg. His abdomen was slightly distended, and abdominal palpation revealed slight diffuse tenderness. Abdominal radiography revealed air/fluid levels. Routine blood tests and tumor markers were normal. Abdominal computed tomography (CT) showed local thickening of the walls and dilation of the small intestine due to intestinal obstruction. Therefore, the patient was diagnosed with small bowel obstruction and underwent emergency hospitalization. Contrast‐enhanced CT was performed on day 1 to identify the causes of intestinal obstruction (Figure 1). An enhanced nodule was detected at the lead point of the obstruction. The inner (mucosa) and outer (muscularis propria and serosa) layers were enhanced, and the middle (submucosa) layer was focally thick. The thickness of the lesion was 5.6 mm, and that of the surrounding normal wall was 3.0 mm. Enhanced CT also showed enlarged lymph nodes near the nodule. We considered elective laparoscopic surgery after decompression of the small bowel. A long intestinal tube was placed to decompress the gastrointestinal pressure during the night of day 1. Roentgenography using a long intestinal tube revealed that the bowel was almost completely obstructed, which led us to suspect the existence of a tumor. Following gastrointestinal decompression, further investigation via single‐balloon enteroscopy was performed transanally on day 4. Enteroscopic images revealed stenosis caused by a circumferential thick and swollen elevated submucosal lesion, which appeared to be covered with non‐neoplastic epithelium. The entire lesion was not observed due to stenosis restricting the passage of the endoscope (Figures 2 and 3). We performed a biopsy and marked the tumor site using the clip‐marking and Indian ink‐injection methods. Pathological tissue specimens contained atypical cells. Based on enteroscopy and pathological findings, we considered that this obstruction was caused by a malignant tumor and required surgical resection. On day 9, laparoscopic small bowel resection with mesenteric lymph node dissection was performed. The surgical view showed a palpable, elastic, hard mass in the jejunum. The lymph nodes that were supposed to flow in (the paraintestinal and intermediate lymph nodes) were also dissected. Macroscopic examination of the resected specimen revealed a poorly circumscribed white tumor of approximately 100 mm in diameter that had caused stenosis (Figure 4a,b). Histologically, hematoxylin and eosin staining showed that the tumor extended from the submucosa to the subserosa (Figure 4c). The tumor contained solid cellular foci of short spindle‐shaped cells and round cells accompanied by mitotic cells (10 cells/10 HPF). Immunochemical staining revealed that the cells were positive for SOX‐10 (diffuse and strong, Figure 4d) and S‐100‐protein (diffuse, Figure S1) and negative for synaptophysin, leukocyte common antigen, cytokeratin (AE1/AE3), cytokeratin 19, desmin, smooth muscle actin, c‐kit, HMB‐45, epithelial membrane antigen, chromogranin, CD56, and CDX2. MIB‐1 (Ki67) was expressed at approximately 10%. The fluorescence in‐situ hybridization study was slightly positive for Ewing sarcoma breakpoint region 1 (ESWR1) gene rearrangements. One swollen lymph node was detected during surgery and was pathologically proven to have metastasized.

These results confirmed the diagnosis of GNET. The patient's postoperative recovery was uneventful, and he was discharged from the hospital 10 days after surgery.

## DISCUSSION

GNET is a rare malignant mesenchymal neoplasm that predominantly occurs in the walls of the small bowel, stomach, or large bowel.^2^ Previously reported symptoms include abdominal pain, anorexia, bleeding, anemia, and bowel obstruction, but they are nonspecific and vary.^3^ There is no preference for sex, and many patients are in their 30s and 40s, ranging in age from 15 to 85 years.^4^ Prior to the accumulation of similar cases and investigations, GNETs have been reported as CCSLGT. CCSLGT, which resembles CCS on microscopy, was first described as a tumor that lacks melanin expression and has a rich presence of osteoclast‐like cells.^5^ CCS is a rare soft‐tissue sarcoma that was first described in 1965.^6^ It is reported that the tumors are slow‐growing, mostly common in the region of the foot and knee, and occur chiefly in young adults. The main characteristic of CCS is the expression of melanin pigments and melanocytic markers.^7^ Subsequently, CCSLGT was described as positive for S‐100 and SOX‐10, but lacking melanocyte‐specific markers.^1^ At the ultrastructural level, they lacked evidence of melanocytic differentiation and showed features of neural differentiation.^1^ Genetically, these tumors were characterized by EWSR1 gene rearrangements similar to those in CCS of the tendons and aponeuroses.^8^ In our case, expression of S‐100 protein and SOX‐10 was observed, while staining for the melanocytic marker HMB‐45 was negative. This is typical for GNETs. Moreover, the tumor exhibited an EWSR1‐ATF1 mutation.

In this case, the small intestinal obstruction was not accompanied by symptoms of shock, irregular vital signs, or intestinal ischemia. For safe laparoscopic surgery, we performed intestinal decompression and endoscopic evaluation before surgery.

After contrast‐enhanced CT and enteroscopy, we listed gastrointestinal stromal tumor, malignant lymphoma, advanced cancer (small intestinal adenocarcinoma), sarcoma, and other malignant tumors as differential diagnoses. The necessity of lymph node dissection is different from that in tumors in the small intestine; small bowel adenocarcinoma requires dissection, GIST does not require dissection, and primary small bowel lymphoma (Stage ⅠE) does not require dissection. Lymph node dissection for stenosis due to benign or systemic malignant disease is unnecessary. The entire tumor was not observed; therefore, it was difficult to distinguish between these diseases. However, the stiffness of the tumor and stenosis were unlikely to be caused by benign diseases in this case. We concluded that the stenosis was caused by a malignant tumor, and lymph node dissection should be accompanied by resection of the primary lesion. CT revealed two enlarged lymph nodes. One of which was pathologically proven to have metastasized. However, one of these was undetectable. We considered that the swelling of the lymph nodes was partially caused by inflammation, which was improved by intestinal decompression. In summary, endoscopic findings confirmed and clarified the diagnosis, which is useful for deciding the form of surgery. In addition, the explanation to the patient would change based on endoscopic findings.

Endoscopic marking is useful in cases in which the lesion is not palpated or lacks serosal findings during surgery. Moreover, abdominal adhesions make it difficult to remove the lesion from the body cavity through a surgical opening. In patients who undergo preoperative enteroscopy, endoscopic marking might be helpful for the detection of lesions under laparoscopic surgery.

Currently, only 99 cases have been reported worldwide.^9^ Only two patients whose primary lesions were in the small intestine, including the patient in this report, underwent enteroscopy before surgery.^10^ To our knowledge, there are no other cases in which a long intestinal tube has been used for gastrointestinal decompression. The enteroscopic examination before surgery could help the preoperative diagnosis; moreover, the location of the tumor could be marked for clear determination, especially in laparoscopic surgery.

In summary, we report the case of a man with GNET presenting with small intestinal obstruction. In most cases of GNET in the small bowels, enteroscopic examination before surgery was not performed. However, enteroscopy is beneficial for diagnosis and treatment. Further accumulation of similar cases would help elucidate the endoscopic findings of GNETs.

## CONFLICT OF INTEREST

The authors declare no conflict of interest.

## FUNDING INFORMATION

None.

## ETHICS STATEMENT

All procedures followed have been performed in accordance with the ethical standards laid down Declaration of Helsinki and its later amendments.

## Supporting information




**Supplementary Figure 1**. S‐100‐protein staining of surgically resected specimen (x400).
