# Supplementary figures and images for: Malignant gastrointestinal neuroectodermal tumor presenting with small intestinal obstruction: A case report
Source: DEN Open. 2022 Apr 10;2(1):e119. doi: 10.1002/deo2.119 (PMC9302053; doi:10.1002/deo2.119)

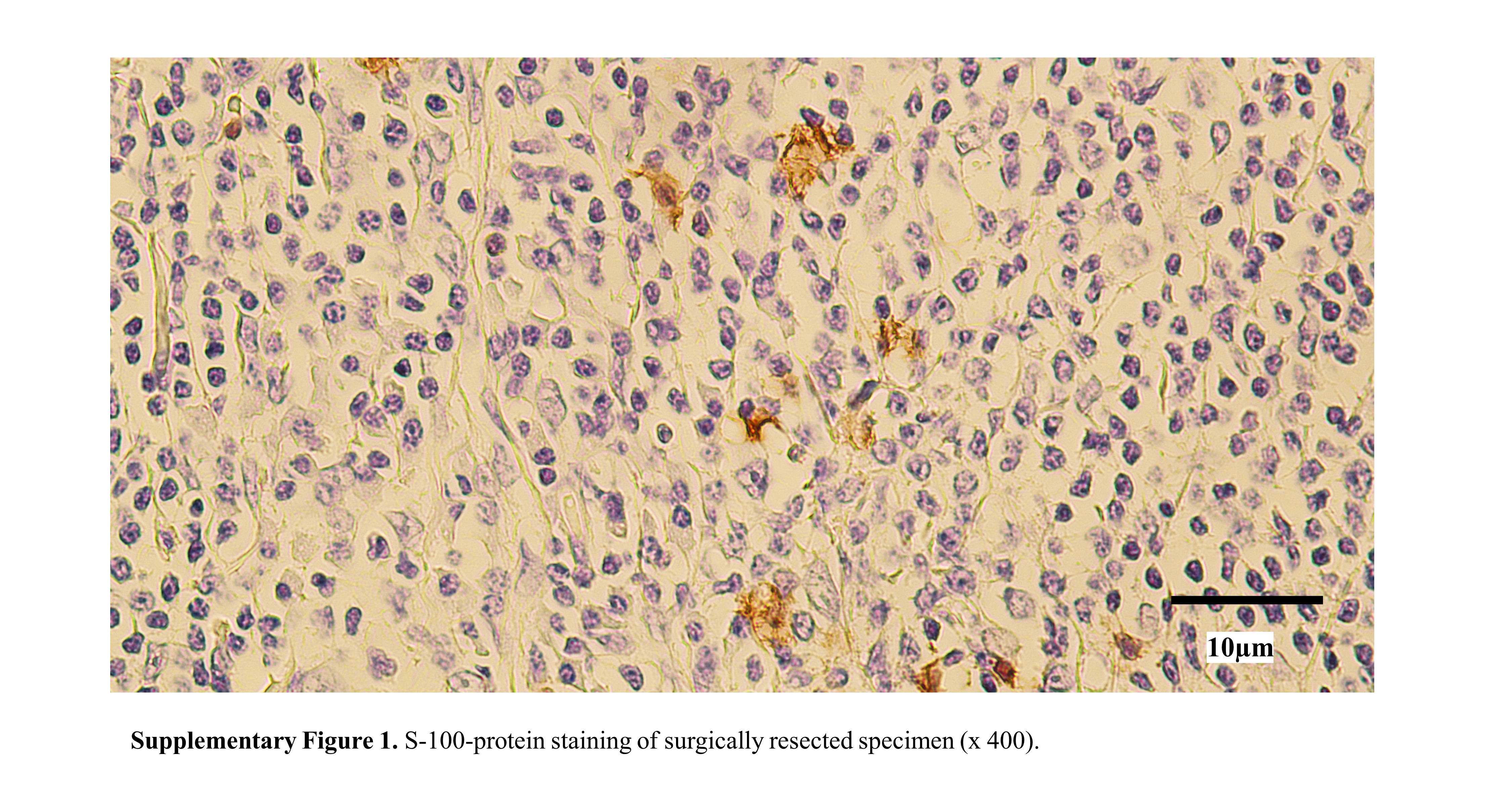

Supplement: Supplementary file 1 — Supplementary Figure 1. S‐100‐protein staining of surgically resected specimen (x400). [file DEO2-2-e119-s001.tif]
